# Classification of Hydroxychloroquine Retinopathy: A Literature Review and Proposal for Revision

**DOI:** 10.3390/diagnostics14161803

**Published:** 2024-08-19

**Authors:** Seong Joon Ahn

**Affiliations:** Department of Ophthalmology, Hanyang University Hospital, Hanyang University College of Medicine, Seoul 04763, Republic of Korea; ahnsj81@gmail.com; Tel.: +82-2-2290-8574

**Keywords:** classification, disease pattern, hydroxychloroquine retinopathy, stage

## Abstract

Establishing universal standards for the nomenclature and classification of hydroxychloroquine retinopathy is essential. This review summarizes the classifications used for categorizing the patterns of hydroxychloroquine retinopathy and grading its severity in the literature, highlighting the limitations of these classifications based on recent findings. To overcome these limitations, I propose categorizing hydroxychloroquine retinopathy into four categories based on optical coherence tomography (OCT) findings: parafoveal (parafoveal damage only), pericentral (pericentral damage only), combined parafoveal and pericentral (both parafoveal and pericentral damage), and posterior polar (widespread damage over parafoveal, pericentral, and more peripheral areas), with or without foveal involvement. Alternatively, eyes can be categorized simply into parafoveal and pericentral retinopathy based on the most dominant area of damage, rather than the topographic distribution of overall retinal damage. Furthermore, I suggest a five-stage modified version of the current three-stage grading system of disease severity based on fundus autofluorescence (FAF) as follows: 0, no hyperautofluorescence (normal); 1, localized parafoveal or pericentral hyperautofluorescence on FAF; 2, hyperautofluorescence extending greater than 180° around the fovea; 3, combined retinal pigment epithelium (RPE) defects (hypoautofluorescence on FAF) without foveal involvement; and 4, fovea-involving hypoautofluorescence. These classification systems can better address the topographic characteristics of hydroxychloroquine retinopathy using disease patterns and assess the risk of vision-threatening retinopathy by stage, particularly with foveal involvement.

## 1. Introduction

Standardizing disease classification is essential for communication among healthcare professionals as it provides common terms that allow for the sharing of standardized information. This is particularly important for hydroxychloroquine retinopathy, an uncommon yet serious condition that lacks adequately described standard terminologies [1,2]. Hydroxychloroquine retinopathy is a retinal toxicity caused by prolonged use of hydroxychloroquine, a medication commonly prescribed for autoimmune diseases such as lupus and rheumatoid arthritis [3,4,5,6,7,8]. The condition can lead to irreversible vision loss if not detected early [9,10,11,12]. Several classification systems have been developed for hydroxychloroquine retinopathy [13]; however, consensus on a scientifically robust classification system has not been reached. Establishing a standardized classification is critical for improving clinical and research communications, ultimately enhancing patient outcomes.

A disease classification system should be systematic, inclusive, practical, and easy to implement. Furthermore, all cases should be confined to mutually exclusive categories within a classification. Specifically, a classification system used for retinal diseases would benefit from addressing the location and severity of the disease, as these characteristics significantly affect visual function in patients [14,15]. Although a few classification schemes based on the distribution of retinal damage and severity have been presented for hydroxychloroquine retinopathy [16,17,18], they have been inconsistent, and therefore, the comparison seems impossible across different studies. Furthermore, several findings from modern retinal imaging studies highlight the limitations of the current classification systems such as discrepancies between imaging modalities [19,20]. Therefore, there is a substantial need for revised classification systems for hydroxychloroquine retinopathy that can be widely and consistently used for diverse cases.

Accordingly, this review aims to summarize the various classification systems used to categorize the patterns of hydroxychloroquine retinopathy and grade its severity as found in the literature. I also examine the limitations of these classifications in light of recent findings from multimodal imaging and studies on the natural progression of the disease. Additionally, we propose revised classification systems to improve the categorization of hydroxychloroquine retinopathy, focusing on both disease patterns and severity.

## 2. Classification Systems of Hydroxychloroquine Retinopathy in the Literature

The understanding and classification of hydroxychloroquine retinopathy have evolved significantly in recent years, particularly with the advances in retinal imaging techniques. Modern classification systems focus primarily on two aspects: the topographical distribution of retinal damage and disease severity staging [13,18,21,22].

### 2.1. Modalities Used for Classification: Advances in Retinal Imaging

Initially, the diagnosis of hydroxychloroquine retinopathy relied heavily on clinical examination such as funduscopy and functional tests such as visual field testing [23,24,25,26]. However, these methods were often insufficient for detecting early and subtle changes [27,28,29]. The introduction of high-resolution optical coherence tomography (OCT) such as spectral-domain OCT and swept-source OCT marked a pivotal advancement, providing high-definition cross-sectional images of the retina. OCT allows for the detailed visualization of retinal layers, enabling the detection of early structural changes before they become apparent on clinical examination [30]. This technology has become a cornerstone in the early diagnosis and classification of hydroxychloroquine retinopathy. Fundus autofluorescence (FAF) imaging further advanced the detection capabilities by highlighting metabolic changes in the retinal pigment epithelium (RPE). FAF can reveal areas of increased or decreased autofluorescence within single image [2,31,32]. This imaging modality has been particularly useful in identifying the extent and pattern of retinal involvement. For example, combined RPE damage is easily identifiable on FAF by detecting hypoautofluroescence. It also aids in the classification of retinopathy into parafoveal, pericentral, and mixed patterns based on the topographical distribution of damage. Thus, the combination of OCT and FAF has provided a more comprehensive understanding of hydroxychloroquine retinopathy, allowing for more accurate classification and staging [33].

In addition to OCT and FAF, multifocal electroretinography (mfERG) has emerged as a valuable tool in the detection of hydroxychloroquine retinopathy. mfERG measures electrical responses from different regions of the retina, providing functional information that complements the structural data from OCT and FAF. This technique can detect localized retinal dysfunction, which is crucial for identifying early retinal toxicity before clinical symptoms arise [34,35]. However, its role in the classification of hydroxychloroquine retinopathy is not fully explored and should be investigated in future studies [36].

### 2.2. Classiciation According to Topographical Distribution

Several classification systems have been proposed to document the distribution of retinal damage. Published studies have converged on describing two distinct patterns of retinopathy: parafoveal and pericentral [37,38,39]. Hydroxychloroquine retinopathy is now recognized to manifest in three distinct patterns based on the location of retinal involvement.

Parafoveal Pattern: This is the classic presentation, characterized by retinal changes occurring 2° to 6° or 8° from the fovea [13,21,22]. It corresponds to the traditional “bull’s eye” maculopathy described in earlier literature. However, it is important to note that with modern screening techniques, retinopathy can be detected before the characteristic bull’s eye appearance becomes visible on fundoscopic examination and needs to be defined using modern imaging modalities such as optical coherence tomography (OCT) and fundus autofluorescence (FAF).

Pericentral Pattern: In some patients, the initial damage occurs in a more peripheral distribution near the major retinal vascular arcades. This predominantly extramacular pattern of damage is referred to as pericentral retinopathy, as opposed to parafoveal retinopathy [39,40]. By definition, this pattern involves retinal changes occurring ≥ 8° from the fovea [17,18]. This presentation underscores the importance of wider imaging or screening that extends beyond the central macula.

Mixed Pattern: Some patients, particularly those with advanced disease, exhibit both parafoveal and pericentral patterns of retinal damage. 

Accordingly, in most studies on hydroxychloroquine retinopathy, the eyes were grouped into two or three groups (parafoveal, pericentral, and mixed) based on the topographical distribution of retinal damage [2], as summarized in Table 1. 

### 2.3. Severity Staging

Severity staging is another crucial aspect of the modern hydroxychloroquine retinopathy classification. This typically involves categorizing the disease into early, moderate, and severe stages based on the extent of retinal damage [17,18]. These classification systems not only aid in accurate diagnosis but also inform prognosis. For example, studies have reported that the progression of retinopathy can be halted by drug cessation if detected before RPE damage (classified as severe retinopathy), emphasizing the critical importance of early detection and stage of retinopathy at the time of diagnosis for future behaviors of retinopathy [17,41].

Although several definitions of severity grades have been proposed in the literature [16,17,18], the majority of studies have classified eyes as having early, moderate, or severe grades based on the extent of outer retinal damages. Early and moderate retinopathies were defined as patchy photoreceptor defects (≤180° around the fovea) and photoreceptor ring (>180°) defects, respectively. Eyes with combined defects in the RPE (observed as hypo-autofluorescence on FAF) were considered to have severe retinopathy [2,17,18,41], as listed in Table 2. 

However, Allahdina et al. suggested a different staging system based on OCT findings as follows: Stage 1, subtle changes confined to the parafoveal region; Stage 2, definite localized changes in the parafovea; Stage 3, extensive parafoveal changes; and Stage 4, foveal involvement [13]. Additionally, Lally et al. proposed another staging system as follows [21]: early, no disruption of parafoveal or foveal ellipsoid zone (EZ); obvious, disruption of parafoveal EZ with intact foveal EZ; and severe, disruption of both foveal and parafoveal EZ (Table 2). 

On the other hand, novel imaging techniques, such as adaptive optics scanning light ophthalmoscopy and OCT retinal thickness deviation map or sequential thickness plot, are showing promise in detecting subtle retinal changes even before they become apparent on conventional imaging modalities [20,42,43,44,45]. These advanced techniques may allow for even earlier staging and intervention in the future, potentially improving long-term visual outcomes in patients receiving hydroxychloroquine therapy.

## 3. Clinical Significance of Classifications for Hydroxychloroquine Retinopathy

### 3.1. Racial Variations in Presentation (Retinopathy Pattern) and Screening Recommendations

The classification of hydroxychloroquine retinopathy patterns has significant implications for screening and management, particularly when considering racial and ethnic variations. Studies have shown that Asian patients are more likely to develop a pericentral pattern of retinopathy compared to other racial groups [38,40]. For instance, the most dominant pattern was pericentral among the Asian populations in previous studies, whereas only 2% of white patients had pericentral retinopathy [38,39,40].

The recognition of pericentral and peripheral patterns of hydroxychloroquine retinopathy has highlighted the critical importance of wide-field screening techniques [17,19,46]. Traditional screening methods that focus solely on the central macula may miss early signs of toxicity in patients with pericentral or peripheral involvement. In contrast, wide-field imaging modalities, such as ultra-widefield fundus autofluorescence and wide-field optical coherence tomography, can provide a more comprehensive view over the wider retinal areas by particularly covering the pericentral areas around major vascular arcades [19,47]. Therefore, these techniques allow for the detection of pericentral retinal changes beyond the central macula, which is particularly important for Asian patients and potentially for other racial groups at higher risk for pericentral retinopathy. Recent studies showed that wide-field screening can help in the early detection of retinal changes before they become clinically apparent or symptomatic [2]. This is crucial because hydroxychloroquine retinopathy is not reversible and early detection is key to preventing permanent vision loss.

This racial variation in retinopathy patterns necessitates tailored screening approaches. Current screening recommendations for hydroxychloroquine retinopathy emphasize the importance of regular, comprehensive evaluations. The American Academy of Ophthalmology (AAO) recommends baseline screening within the first year of starting hydroxychloroquine therapy to rule out pre-existing maculopathy [2,32]. Annual screening should begin after five years of continuous use for patients on acceptable doses and without major risk factors. For patients with significant risk factors (such as high doses, long duration of use, renal disease, or concomitant tamoxifen use), earlier and more frequent screening may be warranted. 

However, for non-Asian patients, it is crucial to perform screening targeting the central macula to sensitively detect parafoveal changes such as standard 6 mm OCT B-scan or 10-2 visual field test [2,32]. These tests should be extended beyond the central macula for detection of pericentral retinopathy. Thus, in Asian patients and potentially in other racial groups at higher risk for pericentral retinopathy, wider tests such as the 30-degree or 9 mm OCT scan, wider field fundus autofluorescence, or 24-2 or 30-2 Humphrey visual field tests are recommended for the specific groups [2,17,40,47]. 

### 3.2. Management Decisions Based on Retinopathy Pattern and Stage

Management decisions for hydroxychloroquine retinopathy should be based on both the pattern and stage of retinopathy. Since retinopathy progresses continuously once it reaches advanced stages, early detection is crucial to minimize further structural progression and visual loss.

For patients with early-stage retinopathy, especially if detected before any RPE loss, cessation of hydroxychloroquine may prevent further progression. Even photoreceptor recovery has been reported after drug cessation at early stages. However, the decision to stop medication should be made in conjunction with the prescribing physician, weighing the risks of retinopathy progression against the benefits of continued therapy for the underlying condition.

In cases of pericentral retinopathy, management may differ slightly from parafoveal cases. Since pericentral changes may not immediately threaten central vision, closer monitoring might be considered before drug cessation, especially if the medication is crucial for managing the patient’s systemic condition [17]. Dose reduction can also be an alternative option to reduce the progression rate, although this has not been proved to be an effective strategy for patients with hydroxychloroquine retinopathy.

For advanced stages of retinopathy, immediate cessation of hydroxychloroquine is typically recommended to prevent further retinal damage. However, it is important to note that some progression may occur even after drug cessation, particularly in advanced cases [17,41].

In all cases, management decisions should be individualized, taking into account the patient’s overall health status, particularly their systemic condition for hydroxychloroquine use; stages and patterns of hydroxychloroquine retinopathy at diagnosis; and the potential risks of vision loss. Regular follow-up examinations, the interval of which might depend on the severity stage, are essential to monitor for any progression, even after drug cessation.

## 4. Limitations of the Suggested Classifications of Hydroxychloroquine Retinopathy

Although the three-pattern and three-stage classification systems have been widely used in previous studies, several findings from modern retinal imaging studies highlight their limitations. First, some classification schemes are inappropriate for classifying pericentral diseases because they focus only on the division of parafoveal diseases [13,21,22]. For example, the definition by Allahdina et al. is more suitable for parafoveal retinopathy, which may threaten the fovea in advanced stages, because the criterion is based on parafoveal or foveal amplitudes on multifocal ERG [22] and the distance from the fovea [13]. Additionally, the severe stages in Lally et al.’s definitions are very difficult to reach (too advanced) in pericentral cases. For example, the cases presented in the top panel of Supplementary Figure S1 show severe, advanced pericentral retinopathy with very extensive RPE defects (hypoautofluorescence on FAF). However, the above criteria consider them to be ‘not severe’ or even ‘early’ cases. In contrast, the parafoveal cases with localized hypoautofluorescence over the parafovea and fovea in the bottom of Figure S1 are all considered to be severe. Therefore, these systems are limited in their ability to grade pericentral retinopathy appropriately [21].

Second, the retinal damage in hydroxychloroquine retinopathy is not confined to the parafoveal or pericentral regions. The fovea, posterior pole other than the parafoveal and pericentral areas, and even the peripheral retina can also be involved, as identified in a recent study using ultra-widefield imaging techniques [19]. From the perspective of disease distribution, these areas need to be addressed when classifying hydroxychloroquine retinopathy. In particular, the fovea, which can be affected by centripetal progression of retinal damage toward the central fovea in advanced diseases from studies on the natural history of hydroxychloroquine retinopathy, should be considered as the topographic location of retinal damage in eyes with advanced diseases [17]. 

Classification of disease severity requires additional information because it relates to foveal involvement. Foveal involvement in retinal damage leads to irreversible vision loss in hydroxychloroquine retinopathy. A few longitudinal studies have shown that the risk of central vision loss differs between parafoveal and pericentral diseases [17,41] owing to differences in the frequency of foveal involvement. This suggests that the current systems of severity classification require further specification regarding the threat or involvement of the fovea. 

Furthermore, the modality used to classify diseases was not uniform among the studies [16,17,38,41]. Imaging techniques that capture images in the retinal plane are ideal for disease classification because they can easily determine disease distribution and severity. For example, FAF imaging can identify the severity of retinopathy based on the degree of damage around the fovea, which can be used to distinguish between early and moderate retinopathies. The OCT B-scans, the most used diagnostic retinal imaging modality, cannot determine the extent of retinal damage (in degrees) across the entire retina from a single image. However, early changes can be subtle or absent in FAF imaging [31]. Furthermore, a few studies have indicated cases with photoreceptor defects greater than 180° on OCT but localized (<180 degrees) hyperautofluorescence on FAF [38]. Thus, identifying the full extent of retinal damage for disease classification is challenging and varies depending on the screening test employed, as demonstrated in previous studies [22,31,46]. 

## 5. Proposed Disease Classification System

To overcome the limitations of the current system of disease classification, we propose a revised system for the classification of hydroxychloroquine retinopathy with respect to topographic distribution and severity staging. 

### 5.1. Classification in Terms of Topographic Distribution

**Figure 1:**
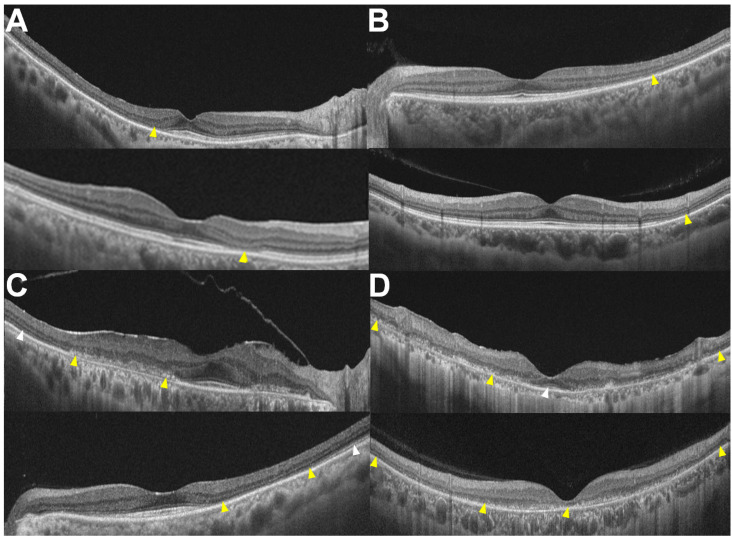
OCT images classified into four patterns: (**A**) parafoveal, (**B**) pericentral, (**C**) combined parafoveal and pericentral, and (**D**) posterior polar, with and without foveal involvement shown at the top and bottom, respectively. Yellow arrowheads indicate outer retinal damage (photoreceptor and/or RPE damage), while white arrowheads indicate relatively intact photoreceptors.

A four-step classification was developed by categorizing the OCT images in a series sorted by increasing severity into four patterns (Figure 1 and Table 3). The area of involvement can be detected sensitively using OCT, whereas FAF sometimes misses early defects [31]. Therefore, OCT was used for the detailed classification of topographic distribution, covering the most vulnerable areas of hydroxychloroquine-induced retinal damage: the parafoveal and pericentral areas.

First, focal susceptibility to retinal damage exists in hydroxychloroquine retinopathy, either parafoveal or pericentral. I noted that eyes with less extensive retinal damage had localized damage in the parafoveal (approximately 500–1500 μm from the foveal center) or pericentral areas. Accordingly, we initially divided the eyes according to the distribution of retinal damage into parafoveal (Figure 1A) and pericentral (Figure 1B) retinopathy, as divided in previous studies [37,38,39]. Some eyes show photoreceptor defects in both parafoveal and pericentral areas (Figure 1C); these are classified as ‘combined parafoveal and pericentral’. In eyes with more advanced disease, the outer retinal defects are not confined to just the parafoveal and pericentral areas but show widespread defects over the posterior pole, extending to the end (margin) of the 30° or 9 mm OCT B-scan, with or without foveal involvement (Figure 1D). From the disease spectrum shown, two distinct manifestations of the disease—parafoveal and pericentral—were retained in the revised classification scheme, while more advanced diseases were detailed as combined and posterior polar as merged forms.

However, this system is complex and may not be practical, particularly for cross-specialty communication in real-world clinical practice. Therefore, for simpler documentation, hydroxychloroquine retinopathy may be separated into parafoveal and pericentral retinopathies. This classification is not strictly based on the topographic location of retinal involvement but rather on the most dominant (extensive or noticeable) manifestations of retinal toxicity, often referred to as the disease epicenter, as many eyes have both presentations, either evidently on conventional retinal imaging or subclinically. This two-step system can highlight the area (or origin) of the most extensive damage, although it may not contain detailed information on the topographic distribution of the involved areas.

### 5.2. Classification According to Disease Severity

**Figure 2:**
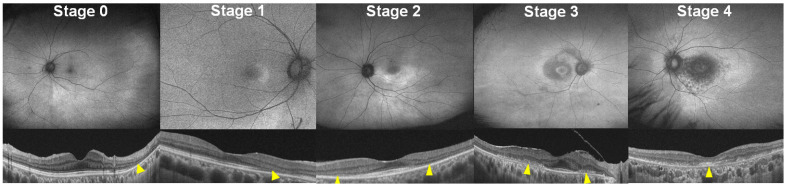
FAF (**top**) and OCT (**bottom**) images classified into five stages (0 to 4) as follows: 0, no definite abnormal FAF; 1, localized hyperautofluorescence; 2, partial (>180° in extent) or full-ring hyperautofluorescence; 3, hypoautofluorescence indicating RPE damage; and 4, hypoautofluorescence with foveal involvement. Yellow arrowheads indicate outer retinal damage (photoreceptor and/or RPE damage).

The current three-stage classification of disease severity in hydroxychloroquine retinopathy can be further refined to specify the foveal involvement and/or disease extent. By specifying foveal involvement and categorizing severe retinopathy based on the presence or absence of foveal involvement, we propose a five-stage disease classification system using FAF findings (Figure 2 and Table 3): 0 (no abnormal findings, hyper- or hypoautofluorescence on FAF), 1 (localized hyperautofluorescence on FAF), 2 (hyperautofluorescence extending over 180° on FAF), 3 (combined RPE damage and hypoautofluorescence on FAF, without foveal abnormality), and 4 (hypoautofluorescence with foveal involvement). Severity staging utilized FAF because it is simple and easy to use for determining stages, displaying disease extent within a single image and simplifying interpretation of combined RPE defects.

This revised staging system may better reflect the risk of vision-threatening retinopathy than current classification systems as it addresses foveal involvement, which is the most significant event in eyes with hydroxychloroquine retinopathy [17]. Furthermore, it uses single modality, FAF, consistently for the staging, and thus, incongruence in disease severity among test modalities can be avoided. 

## 6. Conclusions and Future Directions

I have proposed a revised system for disease classification of hydroxychloroquine retinopathy that addresses the limitations of current systems by integrating recent findings on retinopathy and unifying the descriptions of disease distribution and severity. This system extends applicability to pericentral diseases, previously underrepresented in classification systems. Additionally, this system explicitly specifies the foveal involvement in disease severity, which has significant functional and prognostic value in retinal diseases. 

This systematic approach holds promise for enhancing clinical and research documentation of the disease, facilitating improved communication among ophthalmologists or even between specialties (i.e., rheumatologists and ophthalmologists), through systematic and standardized disease descriptions. However, further validation from a larger cohort of patients with hydroxychloroquine retinopathy from diverse ethnic backgrounds is necessary to establish the reliability of this system. 

The advancement of artificial intelligence (AI) in retinal imaging is poised to revolutionize the classification of hydroxychloroquine retinopathy, as it has in some other retinal diseases such as diabetic retinopathy [48,49,50]. AI algorithms can analyze large datasets of OCT and FAF images to identify patterns and stages of hydroxychloroquine-induced retinal toxicity. These AI tools can classify the severity and progression of retinopathy with high accuracy, enhancing the efficiency of retinopathy screening and ensuring consistent and objective classification criteria. By integrating AI with modern imaging techniques, clinicians can achieve a more precise classification of hydroxychloroquine retinopathy, ultimately leading to better patient prognosis and management.

Another future direction involves the integration of multimodal imaging techniques to provide a more holistic view of retinal damage in hydroxychloroquine retinopathy. Combining OCT, FAF, adaptive optics, and other OCT techniques such as OCT angiography could offer a comprehensive assessment of both structural and functional changes in the retina [51]. This multimodal approach could establish the refined classification systems and understanding of the progression of hydroxychloroquine retinopathy. 

An important consideration in our classification system is the potential variation in prognosis between the pericentral and parafoveal regions, even at the same grade, due to their differing proximities to the fovea. Previous studies have shown that these two patterns of retinopathy usually exhibit similar behaviors, with severe cases often progressing continuously and non-severe cases following a relatively stable course [17,41]. While the suggested classification system does distinguish between pericentral and parafoveal disease, this regional difference is not fully captured within the framework, which is intended to be universally applicable to both patterns. Nevertheless, it is crucial to recognize that regional differences may significantly impact prognosis, particularly in progressive disease, due to varying risks to the fovea. Therefore, more complex criteria incorporating these regional differences might be necessary to account for the different vision-threatening risks and disease prognoses associated with these regions.

Finally, personalized medicine approaches, which will be actively applied to diverse retinal diseases, could revolutionize the management of hydroxychloroquine retinopathy [52]. By considering retinopathy stages, patterns, and individual risk factors such as duration of hydroxychloroquine use, daily dose/body weight ratio, renal diseases, combined use of tamoxifen, and pre-existing retinal conditions [53,54,55], clinicians could tailor monitoring and management strategies to each patient’s unique profile. This individualized approach could optimize the decision on drug cessation or dose reduction and the timing or frequency of monitoring, ensuring appropriate intervention for those at highest risk of progression while potentially reducing unnecessary screenings or avoid cessation of this valuable drug for those with minimal impairment or lower risk of progression. As our understanding of the pathophysiology of hydroxychloroquine retinopathy grows, these personalized approaches could lead to improved overall patient outcomes in terms of systemic conditions and ocular safety and more efficient use of healthcare resources in managing this challenging condition.

## Figures and Tables

**Table 1:** Classifications of hydroxychloroquine retinopathy according to disease pattern.

Classification	Definitions	References
Parafoveal retinopathy	2°–6° from the fovea	[39]
	2°–8° from the fovea	[37,38]
Pericentral retinopathy	Predominantly extramacular pattern of damage near the major retinal vascular arcades (beyond 8° from the fovea)	[37,38,39]
Mixed retinopathy	Both parafoveal and pericentral patterns	[38,39]

**Table 2:** Classifications of hydroxychloroquine retinopathy according to disease severity.

Author	Year	Classifications	References
Marmor	2012	0. Normal on fields, SD-OCT, FAF, mfERG, full-field ERG 1. Patchy damage on fields, SD-OCT, and FAF and parafoveal weakness on mfERG 2. Bull’s eye damage on fields, SD-OCT, and FAF and central subnormal and parafoveal weakness on mfERG 3. Bull’s eye damage + fovea or RPE on fields, SD-OCT, and FAF and amplitudes 25–75% on full-field ERG 4. Diffuse posterior pole damage on fields, SD-OCT, and FAF and amplitudes < 25% on full-field ERG	[22]
De Sisternes et al.	2015	Early as patchy ellipsoid zone damage in the parafoveal region (i.e., areas of damage with parafoveal localization, but not coalesced into a clear ring) Moderate as a clear ring (50% to 100% complete) of damage, but still without RPE involvement observed by SD-OCT, funduscopy, and/or autofluorescence Severe as having RPE damage (by any of the assessment techniques) in the parafoveal bull’s-eye region	[18]
Lally et al.	2016	Early, no disruption of parafoveal or foveal EZ Obvious, disruption of parafoveal EZ with intact foveal EZ Severe, disruption of both foveal and parafoveal EZ	[21]
Allahdina et al.	2019	1. Subtle changes confined to the parafoveal region 2. Definite localized changes in the parafovea 3. Extensive parafoveal changes; and 4, foveal involvement on OCT	[13]

**Table 3:** Revised classifications of hydroxychloroquine retinopathy according to retinopathy pattern and severity.

Criterion	Modalities Used	Classifications	Definitions
Pattern	Optical coherence tomography (OCT)	Parafoveal	2°–6° from the fovea (approximately 500–1500 μm from the foveal center)
		Pericentral	Beyond 8° from the fovea
		Combined parafoveal and pericentral *	Retinal damage involving, but confined to, both parafoveal and pericentral areas
		Posterior polar with or without foveal involvement *	Retinal damage involving wide areas of the posterior pole (extending to the margin of the 30° or 9-mm OCT B-scan), with or without the fovea
Severity	Fundus autofluorescence (FAF)	Stage 0	No abnormal finding, hyper- or hypoautofluorescence on FAF
		Stage 1	Localized hyperautofluorescence on FAF
		Stage 2	Hyperautofluorescence extending over 180° on FAF
		Stage 3	Combined RPE damage, hypo-autofluorescence on FAF, without foveal abnormality
		Stage 4	Hypoautofluorescence with foveal involvement

* May be omitted when considering only the most dominant pattern for classification.

## Data Availability

Data are unavailable due to privacy and ethical restrictions.

## Supplementary Materials

The following supporting information can be downloaded at: https://www.mdpi.com/article/10.3390/diagnostics14161803/s1, Figure S1: Examples of fundus autofluorescence images showing pericentral hydroxychloroquine retinopathy with extensive peripheral involvement (top) and localized parafoveal hydroxychloroquine retinopathy (bottom).
