# Supplementary figures and images for: Classification of Hydroxychloroquine Retinopathy: A Literature Review and Proposal for Revision
Source: Diagnostics (Basel). 2024 Aug 19;14(16):1803. doi: 10.3390/diagnostics14161803 (PMC11353870; doi:10.3390/diagnostics14161803)

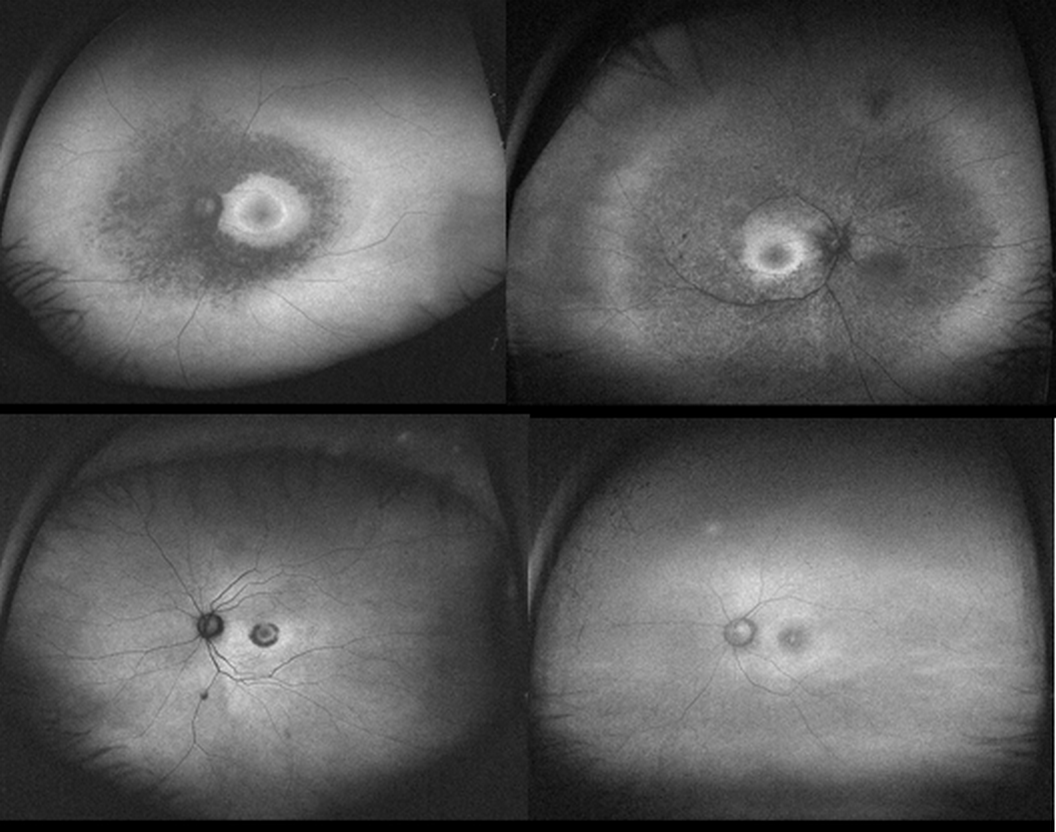

Supplement: Supplementary file 1 [file diagnostics-14-01803-s001.zip › Figure S1.tif]
